# What makes long-term resistance-trained individuals so strong? A comparison of skeletal muscle morphology, architecture, and joint mechanics

**DOI:** 10.1152/japplphysiol.00224.2019

**Published:** 2019-12-24

**Authors:** Thomas M. Maden-Wilkinson, Thomas G. Balshaw, Garry J. Massey, Jonathan P. Folland

**Affiliations:** ^1^Academy of Sport and Physical Activity, Faculty of Health and Wellbeing, Collegiate Campus, Sheffield Hallam University, Sheffield, United Kingdom; ^2^School of Sport, Exercise, and Health Sciences, Loughborough University, Leicestershire, United Kingdom; ^3^Versus Arthritis Centre for Sport, Exercise and Osteoarthritis, Loughborough University, Leicestershire, United Kingdom

**Keywords:** muscle architecture, regional hypertrophy, skeletal muscle, strength training

## Abstract

The greater muscular strength of long-term resistance-trained (LTT) individuals is often attributed to hypertrophy, but the role of other factors, notably maximum voluntary specific tension (ST), muscle architecture, and any differences in joint mechanics (moment arm), have not been documented. The aim of the present study was to examine the musculoskeletal factors that might explain the greater quadriceps strength and size of LTT vs. untrained (UT) individuals. LTT (*n* = 16, age 21.6 ± 2.0 yr) had 4.0 ± 0.8 yr of systematic knee extensor heavy-resistance training experience, whereas UT (*n* = 52; age 25.1 ± 2.3 yr) had no lower-body resistance training experience for >18 mo. Knee extension dynamometry, T1-weighted magnetic resonance images of the thigh and knee, and ultrasonography of the quadriceps muscle group at 10 locations were used to determine quadriceps: isometric maximal voluntary torque (MVT), muscle volume (Q_VOL_), patella tendon moment arm (PTMA), pennation angle (QΘ_P_) and fascicle length (QF_L_), physiological cross-sectional area (QPCSA), and ST. LTT had substantially greater MVT (+60% vs. UT, *P* < 0.001) and Q_VOL_ (+56%, *P* < 0.001) and QPCSA (+41%, *P* < 0.001) but smaller differences in ST (+9%, *P* < 0.05) and moment arm (+4%, *P* < 0.05), and thus muscle size was the primary explanation for the greater strength of LTT. The greater muscle size (volume) of LTT was primarily attributable to the greater QPCSA (+41%; indicating more sarcomeres in parallel) rather than the more modest difference in F_L_ (+11%; indicating more sarcomeres in series). There was no evidence in the present study for regional hypertrophy after LTT.

**NEW & NOTEWORTHY** Here we demonstrate that the larger muscle strength (+60%) of a long-term (4+ yr) resistance-trained group compared with untrained controls was due to their similarly larger muscle volume (+56%), primarily due to a larger physiological cross-sectional area and modest differences in fascicle length, as well as modest differences in maximum voluntary specific tension and patella tendon moment arm. In addition, the present study refutes the possibility of regional hypertrophy, despite large differences in muscle volume.

## INTRODUCTION

Muscular strength is integral to athletic performance (21), helps to reduce injury risk (19) and the likelihood of developing musculoskeletal disorders such as osteoarthritis (84), and also facilitates independence and functional mobility (18, 53) with aging. Participation in resistance training (RT) is well known to increase strength and therefore is widely recommended on an on-going/continuous (i.e., long-term) basis for individuals of all ages as well as numerous patient groups (3, 49, 51, 72). Hence long-term RT individuals are known to be substantially stronger than untrained controls (UT) (9, 56), a functional difference that is often attributed to their larger muscle size [i.e., greater volume or cross-sectional area (CSA) due to hypertrophy]. However, the role of other morphological and mechanical differences that may also influence strength, notably specific tension (i.e., force per unit area), muscle architecture, and joint moment arm, have been poorly documented.

In fact, long-term systematic RT (i.e., multiple years) has been shown to result in substantially greater muscle size compared with untrained controls {+70–76% greater biceps brachii anatomical CSA [ACSA (9, 52)]; +85% greater quadriceps volume (38)}, but whether an increase in muscle size is accompanied by similar, smaller, or no changes in maximum voluntary specific tension (ST) remains unknown. Furthermore, the extent to which increases in overall muscle size (volume) after long-term RT are due to increases in either sarcomeres in parallel (i.e., increased physiological CSA; PCSA) and/or in series (i.e., fiber/fascicle length) has not been examined. Finally, the extent of region-specific hypertrophy, both between constituent muscles and along their length, after long-term RT remains to be elucidated. Therefore, a rigorous assessment of muscle size (ACSA, PCSA, and volume), ST, and architectural contributions to enhanced strength after long-term RT appears warranted.

ST during maximum voluntary contractions (MVCs) is a widely suggested adaptation to RT (24) that encompasses the functional consequences of any changes in neuromuscular activation of the agonist muscle, as well as any changes in intrinsic contractile ST (e.g., perhaps due to a shift in fiber type composition, decreases in antagonist activation, increase in lateral force transmission or reduced fat infiltration) (10). While ST has been quantified using a relatively crude calculation of external force/torque divided by ACSA, a more valid approach involves accounting for antagonist torque and moment arm to calculate agonist muscle force that can be expressed in proportion to PCSA to determine the ST of the agonist muscle. This more rigorous approach has only been used over 9 wk of RT (30) demonstrating an increase in ST of 17%; therefore the ST of individuals who have completed several years of regular systematic heavy RT, and thus the contribution of this variable to their greater strength, remains unknown.

Short-term RT (2–6 mo) appears to result in nonuniform hypertrophy both along and between muscles (25, 44, 61). For example, within the quadriceps numerous studies have found greater hypertrophy of the rectus femoris compared with the vastii (26, 43, 44, 58, 61, 69, 75, 81). Short-term RT studies have also reported the greatest increases in anatomical cross-sectional area (ACSA) to occur at surprisingly diverse points along the muscle: at maximal ACSA (ACSA_max_; 24, 28, 30, 56), in the proximal (63) or distal (26, 35, 58), or even proximal and distal (4, 61) regions of the muscle. These diverse findings could potentially be due to the differences in the prescribed training task or be contraction mode dependent (33, 35, 68) or may in part reflect difficulties in accurately replicating measurement sites along the muscle/limb in studies that typically used a limited number of MRI slices [e.g., 3–7 slices (43, 44, 61)] or ultrasound measures (26, 58, 67), in which case careful description of ACSA along the whole muscle in relation to definitive anatomical landmarks (i.e., the ends of the underlying bone) are required. Moreover, if region-specific hypertrophy resulting from RT does exist it would be expected to be pronounced in long-term RT individuals that exhibit substantially larger muscles; however, this has not been examined.

The structural remodeling of muscle morphology in response to RT can be observed by examining muscle architecture, specifically pennation angle (Θ_P_) and fascicle length (F_L_). Numerous studies have found Θ_P_ to increase after RT (1, 10, 57), and after RT interventions (12, 15, 16, 67); or be higher in resistance-trained versus untrained individuals on a cross-sectional basis (39, 47, 70). An increase in Θ_P_ may facilitate an increase in the contractile material attaching to the tendon/aponeurosis, independent of any change in ACSA. However, the increase in Θ_P_ also has a negative effect on force-generating capacity by reducing the transmission of force between the fibers and the tendon/aponeurosis (8). These contrary effects of Θ_P_ on the force-generating capacity of the muscle are theoretically best reflected by effective PCSA (Q_EFF_PCSA) that accounts for both the number of sarcomeres in parallel and force transmission to the aponeurosis/tendon.

The changes in F_L_ after short-term RT remain controversial with reports of no change in F_L_ [isometric RT (6); or conventional isoinertial RT (lifting and lowering) (14, 26, 29, 30, 80] and increased F_L_ [isometric (65); isoinertial (7, 78)]. One study of long-term heavy RT individuals [RT history: 12.4 ± 5.4 yr (mean ± SD)] observed no difference in F_L_ compared with controls (70). The controversy surrounding the architectural changes, especially F_L_, after RT, could in part be due to heterogeneous architectural changes throughout the muscle after RT (14, 59) in a similar manner, and potentially linked to region-specific hypertrophy. Therefore, comprehensive architectural measurements throughout the muscle may clarify whether F_L_ changes after long-term RT.

Moment arm has been found to have a weak, but significant, association with maximal torque production (17, 77) in untrained controls (74, 79). For some muscles it has been suggested that muscle growth after RT may cause an advantageous increase in the moment arm by positioning the tendon further from the joint center (79). Although the anatomy of the patella and patella tendon wrapping around the distal femur mean that this may be unlikely for the quadriceps, the contribution of any differences in moment arm to the strength in long-term RT individuals compared with untrained individuals is unknown.

The aim of the present study was to determine the factors that explain the greater strength and larger muscle size (volume) of long-term RT individuals (LTT) vs. untrained (UT) individuals. This involved a comprehensive comparison of quadriceps (Q) morphology and mechanics, specifically: measures of muscle size (Q_VOL_, QACSA_MAX_, QPCSA, Q_EFF_PCSA) and regional hypertrophy/muscle mass distribution (between and along the quadriceps muscles) with MRI, agonist muscle ST (accounting for antagonist coactivation, moment arm and Q_EFF_PCSA), muscle architecture (F_L_ and Θ_P_) at 10 sites throughout the quadriceps with ultrasound imaging, and moment arm also assessed with MRI. It was hypothesized that: *1*) the anticipated greater strength of LTT vs. UT would be due to both their greater muscle size (Q_VOL_, QACSA_MAX_, QPCSA, Q_EFF_PCSA) and higher ST; *2*) the greater muscle volume of LTT would be due to higher PCSA rather than greater F_L_ (i.e., sarcomeres in parallel not in series); and *3*) there would be marked regional hypertrophy between and along constituent quadriceps muscles for LTT vs UT.

## MATERIALS AND METHODS

### 

#### Participants and ethical approval.

Sixty-eight young men provided written informed consent before completing this study, which was approved by the Loughborough University Ethical advisory committee and was conducted according to the principles expressed in the Declaration of Helsinki. All participants were healthy and free from musculoskeletal injury. Physical activity levels of all participants were assessed with the International Physical Activity Questionnaire [IPAQ, short format (22)]. The untrained control group (UT, *n* = 52, age 25 ± 2 yr; IPAQ: 2,286 ± 1312 metabolic equivalent min/wk) had no lower-body RT experience for >18 mo. The long-term resistance-trained group (LTT, *n* = 16, age 22 ± 2 yr; IPAQ: 5,383 ± 1495 metabolic equivalent min/wk) reported (via a detailed questionnaire and follow-up oral discussion) systematic, progressive, heavy RT of the quadriceps ~3 ×/wk for ≥3 yr (mean ± SD, 4 ± 1 yr; range, 3–5 yr), involving completion of several knee extensor exercises (e.g., squat, lunge, step-up, knee extension and leg press) within an individual session, and with the primary aim of developing maximum strength. The RT of this group had not been experimentally supervised although some of these participants had received variable coaching (technique and programming) support. Participation in weight classified or predominantly endurance sports was an exclusion criteria to avoid these potential confounders of morphological adaptation. Of the LTT group, resistance training was the only systematic physical activity of 50% (*n* = 8); 38% (*n* = 6) were national-level rugby union players, with the remaining 12% (*n* = 2) competing in powerlifting/body building. Use of androgenic-anabolic steroids was an exclusion criterion for all participants. Many individuals in the LTT group reported regular use of nutritional supplements (e.g., whey protein and creatine).

#### Experimental design.

Participants completed a familiarization session, involving practice of all voluntary contractions performed during subsequent measurement sessions, followed by two duplicate strength measurement sessions separated by 7–10 days. The duplicate strength measurement sessions were typically averaged (for 66 of 68 participants) to enhance the reliability of criterion measurements. Due to availability or injury occurring between sessions two participants completed only one measurement session (both in the LTT group).

Strength measurement sessions were performed at a consistent time of the day for each individual participant, and all sessions started between 1200 and 1900. Participants were instructed not to participate in strenuous physical activity or consume alcohol for 36 h, and to refrain from caffeine consumption for 6 h, before strength measurement sessions. These strength measurement sessions involved a series of incremental warm-up contractions followed by MVCs to establish maximum voluntary torque (MVT) for both the knee extensors and flexors of the dominant limb.

On a separate occasion, musculoskeletal imaging measurements (B-mode ultrasonography and MRI) were performed. Magnetic resonance T1-weighted axial plane images of the thigh were acquired to measure quadriceps muscle size (Q_VOL_ and QACSA_MAX_) with sagittal scans of the knee used to assess patella tendon moment arm (PTMA). Ultrasonographic images were captured at 10 locations throughout the four constituent muscles of the quadriceps (i.e., 2 or 3 locations per muscle) to comprehensively quantify F_L_ and Θ_P_ of the whole muscle group.

#### Torque and electromyographic measurements.

Participants were positioned in an isometric dynamometer with knee and hip angles of 115° and 125° (180° = full extension), respectively. Adjustable straps were tightly fastened across the pelvis and shoulders to prevent extraneous movement. An ankle strap (35 mm width reinforced canvas webbing) was placed ~15% of tibial length (distance from lateral malleolus to knee joint space) above the medial malleolus and positioned perpendicular to the tibia and in series with a calibrated S-Beam strain gauge (Force Logic UK, Berkshire, UK).

The analog force signal was amplified (×370; A50 amplifier, Force Logic UK, Berkshire, UK) and sampled at 2,000 Hz using an A/D converter (Micro 1401; CED, Cambridge, UK) and recorded with Spike 2 computer software (CED). In offline analysis, force signals were low-pass filtered at 500 Hz using a fourth-order zero-lag Butterworth filter (54), gravity corrected by subtracting baseline force, and multiplied by lever length, the distance from the knee joint space to the center of the ankle strap, to calculate torque.

Surface electromyography (EMG) of the hamstring muscles (biceps femoris long head and semitendinosus) was recorded using a wireless EMG system (Trigno; Delsys, Boston, MA). Skin preparation (shaving, abrading, and cleansing with 70% ethanol) was conducted before single differential Trigno Standard EMG sensors (Delsys; fixed 1-cm interelectrode distance) were placed on the biceps femoris long head and semitendinosus at 45% of thigh length above the popliteal fossa. Sensors were placed parallel to the presumed orientation of the underlying fibers. EMG signals were amplified at source (×300; 20- to 450-Hz bandwidth) before further amplification (overall effective gain, ×909), and sampled at 2,000 Hz via the same A/D converter and computer software as the force signal, to enable data synchronization. In offline analysis, EMG signals were corrected for the 48-ms delay inherent to the Trigno EMG system.

#### Knee extension and flexion maximum voluntary contractions.

Following a brief warm-up [3 s contractions at 50% (×3), 75% (×3), and 90% (×1) of perceived maximum], participants performed 3–4 MVCs of the knee extensors for 3–4 s duration interspersed with ≥30 s rest and were instructed to “push as hard as possible.” A horizontal cursor indicating the greatest torque obtained within the session was displayed for biofeedback, and verbal encouragement was provided during all MVCs. The highest instantaneous torque recorded during any MVC was defined as knee extension MVT. Tendon force was calculated as MVT divided by moment arm.

Using the same set-up and warm-up protocol as for the knee extensors, participants performed 3–4 knee flexion MVCs and were instructed to “pull as hard as possible” for 3–4 s and rest for ≥30 s between efforts. A torque-time curve with a horizontal cursor indicating the greatest torque obtained within that session was displayed for biofeedback, and verbal encouragement was provided during all MVCs. Knee flexion MVT was the greatest instantaneous torque achieved during any MVC during that measurement session.

Hamstrings EMG amplitude during knee flexor MVCs was calculated as the root mean square (RMS) of the filtered EMG signal of the biceps femoris long head and semitendinosus over a 500-ms epoch at knee flexion MVT (250 ms either side) and averaged across the two muscles to give HEMG_MAX_. Biceps femoris long head and semitendinosus (antagonist) EMG amplitude during a 500-ms window surrounding knee extension MVT (250 ms either side) was normalized to HEMG_MAX_ from the corresponding EMG sensor. Normalized antagonist EMG amplitude was multiplied by the knee flexor MVT to estimate antagonist knee flexor torque during the knee extension MVCs (assuming a linear relationship between EMG amplitude and torque).

#### MRI measurements of quadriceps muscle size and patella tendon moment arm.

Participants reported to the MRI scanner (1.5-T Signa HDxt, GE) having not engaged in strenuous activity in the prior 36 h and were instructed to arrive in a relaxed state having eaten and drunk normally and sat quietly for 15 min before their MRI scans. T1-weighted MR images of the dominant leg (thigh and knee) were acquired in the supine position at a knee angle of 163° (180° = full extension; due to constraints in knee coil size) and analyzed using OsiriX software (Version 6.0, Pixmeo, Geneva, Switzerland). Using a receiver 8-channel whole body coil, axial images (image matrix 512 × 512, field of view 260 × 260 mm, pixel size 0.508 × 0.508 mm, slice thickness 5 mm, interslice gap 0 mm) were acquired from the anterior superior iliac spine to the knee joint space in two overlapping blocks. Oil-filled capsules placed on the lateral side of the thigh allowed alignment of the blocks during analysis.

**Fig. 1.**
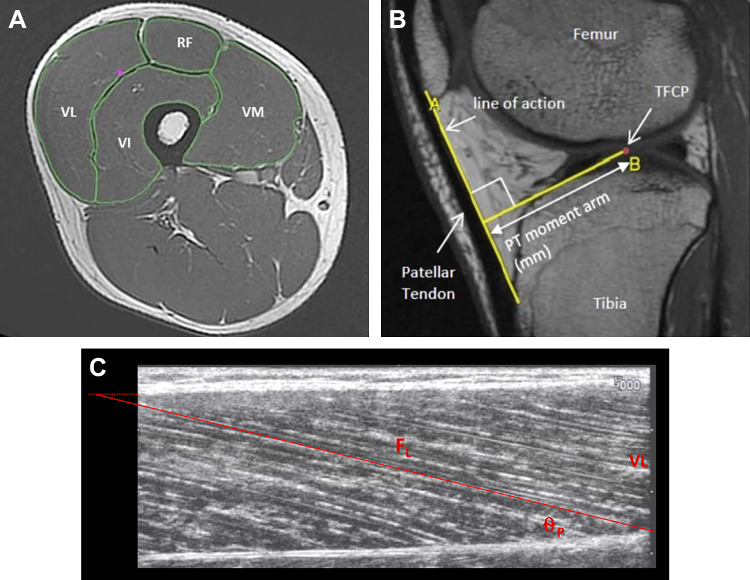
Representative axial MR image of the thigh (*A*); sagittal MRI image of the knee joint (*B*) and muscle architecture (*C*). Patellar tendon (PT) moment arm was defined as the perpendicular distance between the tendon line of action and the tibiofemoral contact point (TFCP). *C* demonstrates muscle architecture measurements of pennation angle (Θ_P_) and fascicle length (QF_L_) from the vastus lateralis.

The quadriceps muscles [vastus lateralis (VL), vastus intermedius (VI), vastus medialis (VM), and rectus femoris (RF)] were manually outlined to determine ACSA in every third image (i.e., every 15 mm; Fig. 1*A*) starting from the most proximal image in which each muscle appeared. This equated to the following number of slices being analyzed per muscle (VM, 23–26; VI, 24–27; VL, 24–27; and RF, 23–26 slices). The volume of each muscle was calculated using cubic spline interpolation of the measured ACSA values/slices (1,000 interpolated points/ACSA values per muscle; GraphPad Prism 6; GraphPad Software) and expressed relative to % femur length. Femur length was defined by the number of slices between the proximal greater trochanter and the knee joint space, multiplied by the slice thickness. For muscle mass distribution, interpolated ACSA for each individual muscle at 5% intervals of femur length were used and expressed relative to ACSA_MAX_. Total quadriceps volume (Q_VOL_) was the sum of the individual muscle volumes. QACSA_MAX_ was calculated by the summation of the maximal ACSA from each individual muscle. Previous data from our group has demonstrated a mean within-participant coefficient of variation for repeat quadriceps muscle volume measurements using the same protocol 12 wk apart with a control group to be 1.7% (11). Inter- and intrarater reliability for Q_VOL_ calculated from the repeated analysis of five MRI scans was 1.2 and 0.4%, respectively.

Sagittal plane images of the knee joint were acquired from the lateral to medial condyles of the femur using an 8-channel knee coil (image matrix 384 × 224, field of view 512 × 512 mm, slice thickness 2 mm, inter-slice gap 0 mm) to determine patella tendon moment arm (PTMA), defined as the perpendicular distance from the patellar tendon line of action to the tibiofemoral contact point (TFCP, the midpoint of the contact between the tibial and femoral condyles; Fig. 1*B*). For maximal voluntary specific tension measurements PTMA length for the MVT specific knee angle was estimated from previously published data fitted with a quadratic function (48) scaled to each participant’s measured moment arm length at 163° as previously (56).

#### Muscle architecture and calculation of PCSA/Q_EFF_PCSA.

Architecture of all four quadriceps constituent muscles (VM, VL, VI, and RF) was examined in detail using B-mode ultrasonography (EUB-8500, Hitachi Medical Systems UK Ltd, Northamptonshire, UK) and a 92-mm, 5–10 MHz linear-array transducer (EUP-L53L). The participant sat in the same isometric dynamometer used for strength measurements while images were captured at rest at 2–3 sites per constituent muscle, for a total of 10 quadriceps architecture measurements sites. Specific sites were over the mid muscle belly (median longitudinal line, i.e., 50% of superficial mediolateral width) at the following percentages of thigh length proximal to the knee joint space: VM 20% (VM_DIS_) and 40% (VM_PRX_), VL and VI at 30% (VL_DIS_, VI_DIS_), 50% (VL_MID_, VI_MID_) and 70% (VL_PRX_, VI_PRX_), and RF 55% (RF_MID_) and 75% (RF_PRX_). The transducer (coated with water soluble transmission gel) was positioned parallel to the long axis of the thigh (femur), and perpendicular to the skin such that an image with the aponeuroses and the perimysium trajectory of several fascicles was clearly identifiable with no visible fascicle distortion at the edge of the image, and with minimal pressure applied on the dermal surface. Video output from the ultrasound machine was transferred to a computer (via an S-video to USB converter) and images recorded using ez-cap video capture software. Images were later imported into public domain software (Image J, v1.48, National Institutes of Health, Bethesda, MD) for analysis.

Θ_P_ was measured as the angle of insertion of the muscle fascicles into the deep aponeurosis, taken as a mean of 3 individual fascicles per ultrasound site. Muscle fascicle length was used as an index of fiber length and sarcomeres in series, and was measured as the length of the fascicular path between the insertions into the superficial and deep aponeurosis; where the fascicular path extended beyond the acquired image the missing portion of the fascicle was estimated by extrapolating linearly the fascicular path and the aponeurosis (48). Due to the long 92 mm ultrasound probe, the extrapolation typically consisted of ≤10% of F_L_. Θ_P_ and F_L_ were averaged over each individual muscle, before calculating an overall quadriceps mean averaged over the four constituents (QΘ_P_ and QF_L_).

PCSA (PCSA) was calculated per constituent muscle as individual muscle volume divided by F_L_ (mean of sites for that constituent), then summed to give quadriceps physiological cross-sectional area (QPCSA)_._ Theoretically PCSA is the best index of contractile material (sarcomeres and cross-bridges) arranged in parallel. To correct for force transmission to the tendon, _EFF_PCSA was calculated as this theoretically best index of muscular force/torque production. Specifically, individual muscle _EFF_PCSA was calculated by multiplying PCSA by cosine of mean Θ_P_ (28), before summing the four constituent muscles to give quadriceps effective PCSA (Q_EFF_PCSA).

#### Calculation of ST.

ST was determined first by the calculation of maximal tendon force; this was done by correcting knee extension MVT for antagonist torque [HEMG at knee extensor MVT normalized to HEMG_MAX_ (i.e., at KF MVT) multiplied by KF MVT] to provide torque from the knee extensors only (66). This knee extensor muscle torque was divided by corrected PTMA (see above) and the subsequent muscle force divided by Q_EFF_PCSA to calculate ST.

#### Statistical analysis.

Muscle strength measured during the duplicate laboratory sessions was averaged to produce criterion values for statistical analysis. An a priori significance level of *P* < 0.05 was set for all statistical tests which were performed using SPSS Version 23.0 (IBM Corp., Armonk, NY). Descriptive data are presented as means ± standard deviation (SD) and percentage differences between groups calculated from group means. The influence of group (UT, LTT) on all muscle architecture and muscle size variables was examined by independent *t*-tests. To examine if the architectural differences between the groups varied with constituent muscle, a 4 × 2 ANOVA [constituent muscle (VL, VM, VI, RF) × group (LLT, UT)] was performed, and if interaction effects were found, then post hoc analysis (pairwise ANOVA contrasting only two muscles) was also performed. Effect size (ES) for absolute difference data was calculated as previously detailed for between-subject study designs (50) and classified as follows: <0.20 = “trivial,” 0.20–0.49 = “small,” 0.50–0.79 = “moderate,” or ≥0.80 = “large.” *P* values were corrected for multiple tests using the Benjamini–Hochberg procedure (13) with a false detection rate of 5%, and significance was defined as adjusted *P* < 0.05. For the whole cohort (i.e., data pooled from both LTT and UT groups, *n* = 68) the relationships between musculoskeletal variables and MVT were first assessed with independent Pearson's product- moment correlations, and then stepwise multiple regression analysis was performed, with only the significant predictors entered into the model.

## RESULTS

### 

#### Participant characteristics and strength.

LTT were taller and heavier than UT (183 ± 6 vs. 176 ± 2 cm; 91 ± 10 vs. 73 ± 10 kg; both *P* < 0.001). MVT was 60% greater in LTT than UT (388 ± 70 vs. 245 ± 43 N·m; *P* < 0.001, ES = 2.5).

#### Total quadriceps and constituent muscle size, and muscle mass distribution between and along the quadriceps muscles.

**Fig. 2.**
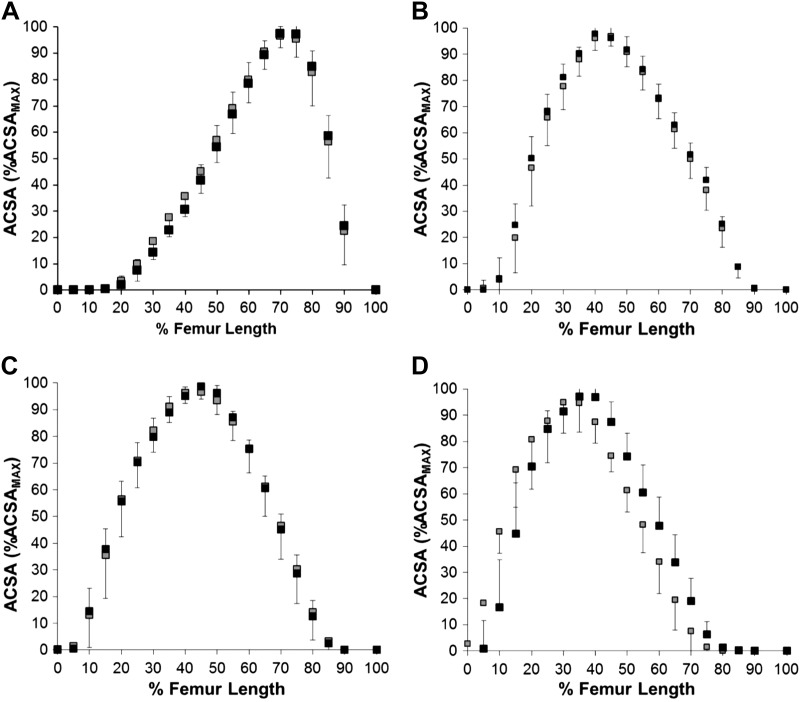
Muscle mass distribution (% of ACSAmax) along the femur (at 5% increments from proximal (0%) to distal (100%)) in untrained men (UT, gray squares; *n* = 52) and long-term resistance-trained men (LTT, black squares ; *n* = 16) for the constituent quadriceps muscles: vastus medialis (*A*), vastus intermedius (*B*), vastus lateralis (*C*), and rectus femoris (*D*). Data are means ± SD. There were no differences between groups for muscle mass distribution (% of ACSAmax) for any muscle or 5% increment along the femur (all adjusted *P* ≥ 0.21). ACSA, anatomical cross-sectional area.

Q_VOL_ was 56% greater in LTT than UT (*P* < 0.001; ES = 3.7), QACSA_MAX_ was 50% greater (*P* < 0.001, ES = 3.3) and Q_EFF_PCSA 41% greater in LTT compared with UT (*P* < 0.001, ES = 4.1). LTT had greater volume of all the individual constituent muscles of the quadriceps (54–58%, *P* < 0.001, ES = 2.3–3.7; Table 1). Likewise, LTT had greater ACSA_MAX_, PCSA, and _EFF_PCSA of all the individual constituent muscles of the quadriceps (ACSA_MAX_, 46–52%, all *P* < 0.001, ES = 1.9–2.9; PCSA, +39–45%, all *P* < 0.001, ES = 1.9–2.6; _EFF_PCSA, +38–44%, all *P* < 0.001, ES = 2.2–2.7) than UT. However, the proportional volume, and ACSA_MAX_, of the individual constituent muscles (to total quadriceps muscle volume and QACSA_MAX_, respectively) were similar for LTT and UT (*P* = 0.56–0.94; volume data shown in Table 1) and the percentage of femur length where ACSA_MAX_ of each constituent muscle occurred was also similar for both groups (VM: 28% vs. 29%; VI: 58% vs. 58%; VL: 57% vs. 56% and RF: 68% vs. 68% femur length for UT and LTT, respectively; *P* = 0.26–0.80; Fig. 2). To further assess regional hypertrophy, the relative distribution of muscle mass along the thigh was examined by plotting relative ACSA (%ACSA_MAX_) against femur length for each constituent muscle (Fig. 2). No differences in relative ACSA were observed between UT and LTT at any position along the femur for any of the constituent muscles (adjusted *P* > 0.21).

**Table 1.** Quadriceps muscle size indices, individual constituent muscle volumes and proportional volumes of untrained (UT) and long-term resistance-trained (LTT) men

Muscle and Size Variable	UT (*n* = 52)	LTT (*n* = 16)	%Difference	Effect Size
Quadriceps				
Q_VOL (_cm^3^)	1,838.2 ± 262.9	2,881.9 ± 308.1*	56	3.7
QACSA_MAX_ (cm^2^)	86.2 ± 11.2	135.0 ± 15.0*	50	3.3
QPCSA (cm^2^)	174.4 ± 19.8	245.7 ± 16.8*	41	3.9
Q_EFF_PCSA (cm^2^)	167.7 ± 18.8	236.8 ± 15.1*	41	4.1
Individual muscle volume (cm^3^)				
VM	441.4 ± 67.8	691.2 ± 87.0*	57	3.2
VI	546.9 ± 104	846.4 ± 124.0*	55	2.6
VL	609.8 ± 98.4	964.3 ± 90.6*	58	3.8
RF	240.2 ± 46.7	374.6 ± 72.0*	56	2.3
Proportional muscle volume (%Q_VOL_)				
VM	24.0 ± 1.7	24.1 ± 1.9	0	0.0
VI	29.7 ± 2.8	29.3 ± 1.6	1	−0.2
VL	33.2 ± 2.6	33.6 ± 2.3	1	0.2
RF	13.1 ± 1.8	13.0 ± 1.8	1	−0.1

Data are means ± SD, Q_VOL_ = quadriceps volume; QACSA_MAX_ = sum of maximal anatomical cross-sectional areas from individual muscles; QPCSA = quadriceps physiological cross-sectional area; Q_EFF_PCSA = effective physiological cross-sectional area; VM = vastus medialis; VI = vastus intermedius; VL = vastus lateralis; RF = rectus femoris;

* indicates adjusted *P* < 0.01

#### Muscle architecture.

**Fig. 3.**
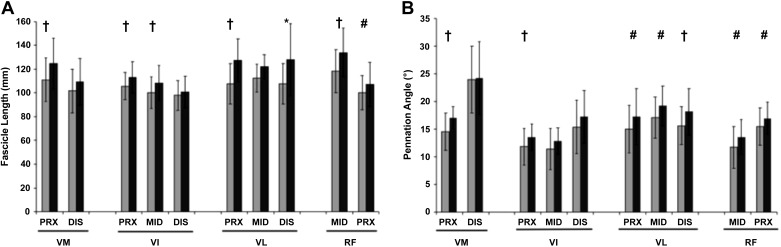
Differences in fascicle length (*A*) and pennation angle (*B*) between untrained (UT) men (gray bars; *n* = 52) and long-term resistance-trained men (LTT, black bars; *n* = 16) at two or three sites of each of the constituent quadriceps muscle. VM, vastus medialis; VI, vastus intermedius; VL, vastus lateralis; RF, rectus femoris; PRX, proximal; MID, middle; DIS, distal. Data are means ± SD. Symbols indicate adjusted *P* values: *P < 0.01, †*P* < 0.05; #tendency for a difference *P* = 0.05–0.07.

QF_L_, based on the mean of 10 sites, was 11% greater in LTT than UT (*P* < 0.001, ES = 1.2; Table 2), and mean F_L_ of each individual muscle was longer (VM: +12%, ES = 0.7; VL: +13%, ES = 1.0; and RF: +12%, ES = 0.8; all *P* < 0.05) or showed a tendency to be longer (VI: +7%; *P* = 0.06, ES = 0.8) for LTT than UT. The outcome of the ANOVA revealed a constituent muscle (VL, VM, VI, RF) × group (LTT, UT) interaction effect (i.e., bigger differences between groups for some muscles than others; *P* = 0.03), and post hoc analysis showed larger differences between UT and LTT in the VM, VL, and RF compared with VI (pairwise ANOVA with only two muscles; group × muscle interaction; all *P* ≤ 0.008). Considering the specific measurement sites, 6 of 10 sites showed greater F_L_ of LTT vs. UT (VM_PRX_, VI_PRX,_ VI_MID_, RF_MID_, VL_DIS_, and VL_PRX_ sites; all *P* < 0.001), with a tendency to be longer for RF_PRX_ (*P* = 0.06) and no differences at the remaining three measurement sites (all *P* > 0.15; Fig. 3*A*).

**Table 2.** Muscle architecture variables, fascicle length (F_L_) and angle of pennation (*Θ_P_*), for untrained (UT) and long-term resistance-trained (LTT) men

Variable	Muscle	Sites Measured	UT (*n* = 52)	LTT (*n* = 16)	%Difference	Effect Size
F_L_ (mm)						
	Q	10	106.4 ± 9.0	118.0 ± 10.0*	11	1.2
	VM	2	104.6 ± 16.4	117.1 ± 17.4†	12	0.7
	VI	3	100.9 ± 8.1	107.5 ± 7.8#	7	0.8
	VL	3	111.1 ± 11.5	125.7 ± 16.8†	13	1.0
	RF	2	109.0 ± 14.8	121.6 ± 17.8†	12	0.8
Θ_P_ (mm)						
	Q	10	15.4 ± 2.9	17.3 ± 2.0*	13	0.7
	VM	2	19.2 ± 3.9	20.8 ± 3.4	8	0.4
	VI	3	12.9 ± 2.6	14.5 ± 2.2#	13	0.7
	VL	3	15.9 ± 2.6	18.2 ± 3.3†	15	0.8
	RF	2	13.5 ± 2.5	15.6 ± 2.4†	16	0.9

Data are means ± SD. Quadriceps and individual constituent muscle values are based on the mean of ten or two/three sites, respectively. Q = mean quadriceps, VM = vastus medialis, VI = vastus intermedius, VL = vastus lateralis, RF = rectus femoris, Θ_P_ = angle of pennation, F_L_ = fascicle length_._ Adjusted *P* values are indicated by:

* *P* < 0.01,

† *P* < 0.05;

# tendency *P* = 0.05–0.07.

QΘ_P_ was 13% greater in LTT than UT (*P* < 0.001, ES = 0.7; Table 2), and reflected a greater mean Θ_P_ in the VL (15%, *P* = 0.02, ES = 0.8) and RF (15.5%, *P* = 0.01, ES = 0.9) but not the VM (9%, *P* = 0.21, ES = 0.4) or VI (13%, *P* = 0.07, ES = 0.7). There were no group × constituent muscle interactions (*P* = 0.826). LTT had greater Θ_P_ than UT at 3 of 10 sites (VM_PRX_, VI_PRX_, VL_DIS;_ *P* < 0.05), with a tendency to be greater observed at four further sites (VL_PRX_, VL_MID_ and both RF sites; adjusted 0.05 ≤ *P* ≤ 0.07; Fig. 3*B*).

#### Patella tendon moment arm (PTMA) and maximum voluntary specific tension (ST).

**Fig. 4.**
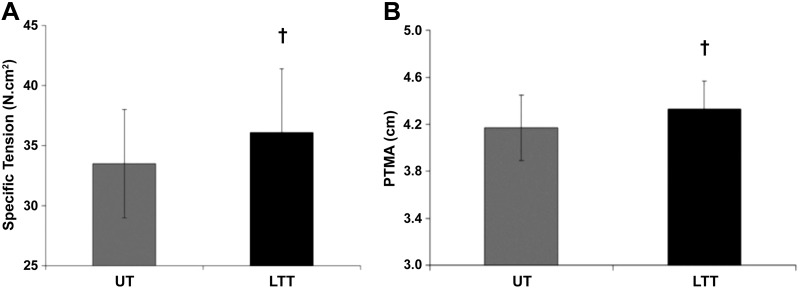
Maximal voluntary specific tension (ST) (*A*) and patella tendon moment arm (PTMA) (*B*) in untrained (UT; gray bars, *n* = 52) and long-term resistance-trained (LTT; black bars, *n* = 16) individuals. †Adjusted *P* < 0.05.

LTT had a 4% greater PTMA than UT (4.17 ± 0.28 cm vs. 4.33 ± 0.24 cm; *P* = 0.03; ES = 0.6: see Fig. 4*B*). However, when normalized to participant’s height, there was no difference in PTMA between groups (PTMA/Height ratio: UT, 0.0237 ± 0.0017 vs. LTT, 0.0236 ± 0.0009; *P* = *0.92;* ES = 0.2). Tendon force was 54% greater in LTT than UT (5,576 ± 905 N vs. 8,564 ± 1,410 N; *P* < 0.001, ES = 2.6). There was 8% greater ST of the quadriceps in LTT than UT (33.3 ± 4.5 N·cm^2^ vs 36.1 ± 5.3 N·cm^2;^ *P* = 0.04, ES = 0.6; Fig. 4*A*) when accounting for antagonist coactivation, corrected PTMA, and Q_EFF_PCSA.

#### Factors that explain the greater strength and muscle mass (volume) of long-term RT individuals.

**Fig. 5.**
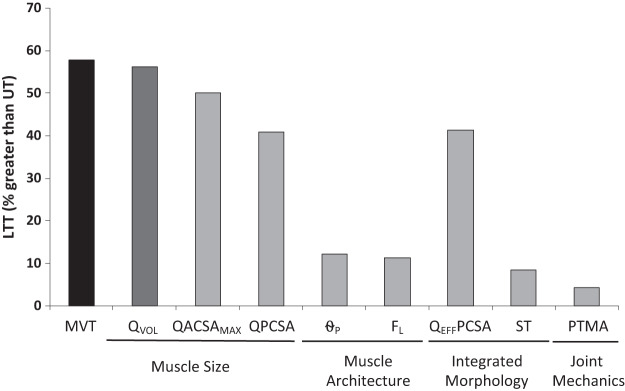
Musculoskeletal variables that appear to contribute to the greater strength and larger muscle volume of long-term resistance-trained (LTT) compared with untrained (UT) men. Data are percentage differences in group mean values for maximal voluntary torque (MVT), quadriceps volume (Q_VOL_), sum of maximal anatomical cross-sectional area (QACSA_MAX_), quadriceps physiological cross-sectional area (QPCSA); quadriceps effective physiological cross-sectional area (Q_EFF_PCSA), mean quadriceps angle of pennation (QΘ_P_), mean quadriceps fascicle length (QF_L_), maximum voluntary specific tension (ST), and patella tendon moment arm (PTMA) between untrained and long-term resistance-trained participants.

**Fig. 6.**
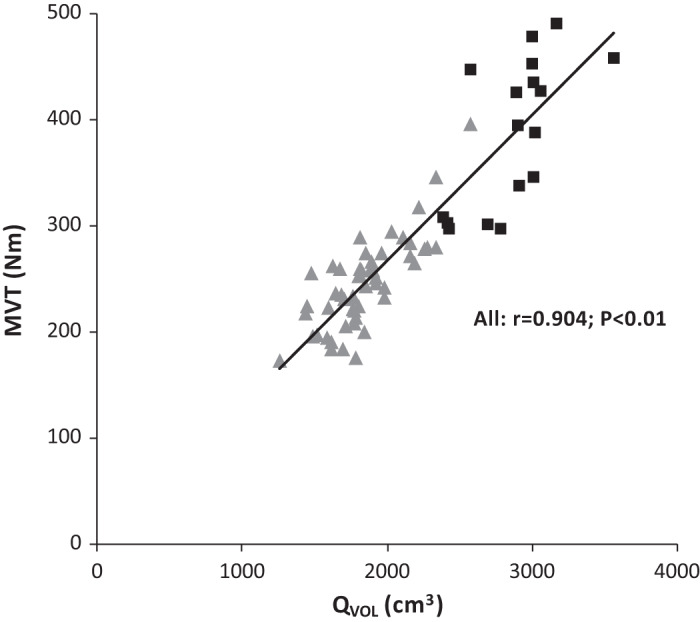
Scatterplot of the relationship between maximal voluntary torque (MVT; N·m) and quadriceps volume (Q_VOL_; cm^3^) in untrained (UT; *n* = 52: triangles) and long-term resistance-trained (LTT; *n* = 16: squares) individuals. Regression line is for all participants(*r* = 0.90; *P* < 0.01).

The difference in strength between LTT and UT (+60%) in comparison to the differences between the groups in a range of underpinning musculoskeletal variables, specifically those variables that were each significantly greater in LTT than UT, are shown in Fig. 5. Of the musculoskeletal variables, the largest differences were in the muscle size indices (Q_VOL_ +56%; QACSA_MAX_ +50%) which therefore provide the primary explanation for the greater strength of LTT. This greater muscle size of LTT in combination with a more modest difference in QΘ_P_ (+12%) resulted in a difference in Q_EFF_PCSA (+40%), which alongside other smaller contributions from ST (+8%) and moment arm (+4%) appears to explain the strength difference. The greater muscle volume of LTT vs. UT (Q_VOL_ +56%) appeared to be primarily due to increased QPCSA (+41%) with a much smaller contribution of QF_L_ (+11%; Fig. 5). Bivariate correlations for the whole cohort (i.e., both groups, *n* = 68) were found between all musculoskeletal variables and MVT [Q_VOL_ *r* = 0.90 (Fig. 6); QACSA_MAX_ *r* = 0.87; Q_EFF_PCSA *r* = 0.87; QΘ_P_ *r* = 0.47; QF_L_ *r* = 0.61; ST *r* = 0.56; PTMA *r* = 0.41; all *P* < 0.01]. Stepwise multiple regression analysis revealed that the only variable to contribute to the explained variance in MVT was Q_VOL_ (*R*^2^ = 0.81; *P* < 0.001).

## DISCUSSION

The aim of the present study was to determine the musculoskeletal factors that explain the greater strength and larger muscle size (volume) of long-term RT individuals vs. untrained individuals. Previous RT studies have typically been short-duration interventions or examined a limited range of musculoskeletal factors, and thus our knowledge of the adaptations to prolonged RT have been limited. In accordance with our first hypothesis the greater muscle strength of LTT (+60%) was accompanied by both a greater quantity of skeletal muscle and higher ST. However, the differences between LTT vs. UT for the indices of muscle size (e.g., ranging from volume +56% to Q_EFF_PCSA 41%) were substantially larger than was the case for ST (+8%), or in fact PTMA (+4%), and thus muscle size was the primary explanation for the greater strength of LTT. For the second hypothesis, the greater Q_VOL_ (+56%) of LTT was due primarily to enhanced QPCSA (41%), indicating more sarcomeres in parallel, although we also found convincing evidence for greater QF_L_ (+11%), indicating a modest difference in sarcomeres in series. Finally, despite the large differences in Q_VOL_, and contrary to our third hypothesis, we found no evidence for regional hypertrophy/muscle mass distribution between or along the constituent quadriceps muscles.

The difference in MVT of LTT vs. UT in the current study was substantial (+60%), but somewhat lower than observed in one previous study [+77% (70)]. The greater MVT of LTT was accompanied by both a greater quantity of skeletal muscle and higher specific tension, although it was clear from the magnitude of the differences that the indices of muscle size (e.g., volume +56%, Q_EFF_PCSA +41%) were substantially larger than was the case for ST (+8%), or in fact PTMA (+4%), and thus muscle size was the primary explanation for the greater strength of LTT. The importance of muscle volume for strength was reinforced by our regression analysis of the whole cohort that found muscle volume was the only determinant of MVT, alone explaining 81% of the variance in strength. Several other studies have found substantially greater muscle size of long-term resistance-trained participants [70% to 86% (9, 37, 46, 49)], but none have previously examined maximum voluntary specific tension to investigate the contribution of force per unit area to the enhanced strength of LTT.

We found modest differences in specific tension (+8%), even after the average 4 yr of regular, heavy RT of LTT. While no previous studies have examined the specific tension of LTT individuals, after short-term (9 wk) RT maximum voluntary specific tension has been reported to increase by 20% (30), which is clearly somewhat contrary to the more modest 8% difference we have found for LTT vs. UT. However, it is notable that Erskine et al. (30), reported average isometric strength gains ~2-fold greater than we have found [31% vs. 11.5–18.2% (31, 32)], with almost identical training regimes and the same number of training sessions, and this discrepancy likely explains the large increase in specific tension they have reported. Nonetheless, numerous short-term RT studies have shown greater increases in strength/force than cross-sectional area, indicating an increase in the specific tension (23, 27, 29, 42, 45, 61, 69, 83). Increased specific tension could be attributable to changes in neuromuscular activation [e.g., increased agonist activation (10, 60)] or an increase in the intrinsic contractile specific tension, perhaps due to a shift in muscle fiber phenotype (20) or alterations in muscle architecture (24). Moreover, the modest difference we have found in specific tension after LTT suggests that increases in specific tension that occur with RT may be relatively limited, and thus the underpinning mechanisms for increased maximum voluntary specific tension (i.e., increased agonist neuromuscular activation or intrinsic contractile specific tension) are also relatively small.

The larger volume of muscle of LTT was primarily due to their greater PCSA (+41%; i.e., sarcomeres in parallel) rather than QF_L_ (+11%; i.e., sarcomeres in series). To our knowledge this is the first report to quantify the contribution of these different aspects of muscle morphology to the enhanced muscle mass of substantially hypertrophied human muscle, and it is clear that muscle growth primarily occurs due to an increase in the contractile material arranged in parallel with a smaller contribution from increased sarcomeres in series. To provide a comprehensive assessment of quadriceps muscle architecture we measured Θ_P_ and F_L_ at 10 sites within the quadriceps, which revealed LTT to have a greater QΘ_P_ (+13%) and QF_L_ (11%) than UT. A greater QΘ_P_ facilitates the attachment of more contractile material, and thus the application of more force, to the tendon/aponeurosis [i.e., as reflected by PCSA (40, 45, 47, 61)], independently from any increase in muscle ACSA or volume, although force transmission to the tendon is increasingly compromised (according to the cosine of Θ_P_). Overall a greater QΘ_P_ is thought to be beneficial for isometric force production up to an optimum angle of 45° (8). Resistance-trained individuals/bodybuilders have previously been found to have much higher Θ_P_ in both the triceps brachii [33° vs. 15°; +120% (47)], mid-point vastus lateralis (20.4° vs. 15.5°; +31% (39)], and medial gastrocnemius [24.6° vs. 18.4°; +34% (39)], which are clearly a larger difference than we found in the present study (QΘ_P_: +11%). This contrast may indicate an anatomical specificity to muscle architectural changes after RT or site-specific differences. Furthermore, the findings of the present study are surprisingly similar to the increases in Θ_P_ observed following short-term lower body RT (2, 10, 26, 35), perhaps suggesting that changes in lower body Θ_P_ may not continue to adapt with prolonged RT and could predominantly occur in the early phase of a training program (i.e., first 3 mo).

The possibility of F_L_ increases after RT, largely based on short-term RT studies, has been controversial (7, 16, 26, 29, 30, 64, 78, 82). Using architecture measurements at 10 sites throughout the quadriceps we found the LTT group to have an 11% greater QF_L_ compared with UT. One previous study of LTT vs. UT reported no differences between their groups (39); however, they assessed F_L_ at only one site, equivalent to the VL_MID_ site of our experiment, where we also observed no differences between LTT and UT (Fig. 3*A*). In contrast, we found a clear difference for 3 of 4 of the individual muscles (VM, VL, and RF), a tendency for a difference in the fourth (VI), and over the whole muscle group QF_L_ showed a highly significant difference with a large effect size (+11%, *P* < 0.01 ES 1.2). We also found quantitative evidence for a training group (LTT vs. UT) by constituent muscle interaction for F_L_, demonstrating inhomogeneous adaptations to LTT. Thus it seems likely that the regional variability in F_L_ changes, the error associated with a single measurement site, the differences in the mode of resistance training used and the short duration of previous reports contribute to the equivocal findings in the literature (34). The current study using a comprehensive assessment at 10 sites throughout the quadriceps muscle group indicates that QF_L_ does increase with prolonged RT. Interestingly, based on geometric modeling it has recently been argued that relatively modest changes in F_L_ can have disproportionately large effects on ACSA and muscle volume (46). In essence, longer (extended) fascicles due to the addition of sarcomeres in parallel appears to result in a disproportionately larger increase of sarcomeres in parallel and therefore could be a key explanation for the differences in muscle size (ACSA, PCSA, and volume) we have observed.

While Θ_P_ did not show such strong evidence for inhomogeneous adaptations to LTT (no training group × muscle interaction effect) there were a range of differences when comparing the four constituent muscles (Θ_P_ 8–15%; F_L_ 6–13%). Therefore, this study further highlights the need for multiple sites to comprehensively quantify architectural differences or changes after training as single sites may be difficult to replicate (36) and as seen in the present study and others, a single site measurement similar to VL_MID_ is not reflective of overall architecture differences across the quadriceps muscle group following RT (26, 35).

Despite the 56% greater muscle volume of LTT vs. UT we found no evidence for regional hypertrophy either between the constituent quadriceps muscles or along their length. Previous short-term RT studies, documenting relatively limited hypertrophy, have, however, repeatedly reported nonuniform regional hypertrophy, both between and along the individual quadriceps muscles, although curiously the pattern of regional hypertrophy has been surprisingly diverse [i.e., which muscles and locations had the greatest hypertrophy (26, 35, 37, 43, 44, 57, 58, 61, 69, 75, 76)]. In the current study, we scanned the entire length of the thigh to accurately identify the ends of the bone and subsequently define the precise position of each of a large number of axial images (slices per muscle: VM, 23–26; VI, 24–27; VL, 24–27; RF, 23–26) relative to those absolute landmarks to carefully quantify regional differences in muscle size. In addition, we recently found a mean within-participant coefficient of variation for repeat quadriceps muscle volume measurements using the same protocol 12 wk apart with a control group to be 1.7%, indicating the reliability of our measurements (11). In contrast, previous studies typically used a small number of slices and positioned slices based on relatively imprecise surface anatomical measurements. Therefore, previous reports of regional hypertrophy may have been confounded by the inconsistent location of the images. Alternatively, as the LTT individuals in the current study had been doing a range of different training practices it is conceivable that this may have resulted in diverse individual hypertrophic responses that cumulatively canceled out and led to no overall regional hypertrophy. However, inspection of the variability (between participant standard deviation) indicates that the proportional size of the individual quadriceps’ muscles (Table 1) and distribution of muscle mass along the femur (Fig. 3) were no more variable for LTT than UT groups. In summary, given the careful methods and large difference in muscle volume in the current study without any evidence for regional hypertrophy it seems likely that this phenomenon may have been overestimated by previous studies.

In addition to morphological changes in the muscle, joint mechanical properties such as PTMA may make a small contribution to maximal torque production (17, 77). In the present study, PTMA was 5% greater in LTT compared with UT. In other muscle groups it has been suggested that muscle hypertrophy may result in biomechanically advantageous increases in leverage of muscular force application (5, 73, 74, 79). However, for the quadriceps the anatomy of the patella and patella tendon wrapping around the distal femur mean that this is unlikely to be the case. In addition, when PTMA was normalized to height there was no difference between the groups indicating that the 4% greater height of LTT group was in large part responsible for their greater PTMA.

There are a number of limitations within the current study that should be recognized. Although the current cross-sectional study design provided a pragmatic approach to examining the substantial adaptations that occur after LTT, due to the cross-sectional nature of the current study and the extensive, retrospective RT background (mean 4 yr RT) of these participants we have relatively limited information regarding their exact training (e.g., precise loads, types of contractions, periodization). Nonetheless these participants all had the primary goal of increasing maximum strength, and were demonstrably stronger than controls (+60%), and we excluded participants involved in activities (e.g., weight category and endurance sports) that might compromise morphological adaptations to RT. A repeated-measurement design on the same participants before, potentially during, and after a prolonged period of RT is clearly a stronger design. Although this approach would be practically challenging, there are very few supervised RT studies of ≥6 mo duration; it would facilitate an in-depth examination of the time course of adaptations to prolonged RT and could be informative for a number of the measures investigated in the current experiment (e.g., specific tension, architecture, regional hypertrophy). The acquisition of clear T1 MR images along the whole thigh (~25 min) is not compatible with measurements during contraction, and in our experience, it is also challenging to record clear ultrasound images of all the constituent muscles during MVCs (55). Thus, the imaging measurements of muscle size, architecture, and moment arm within the current experiment were made at rest to facilitate precise measurements. In addition, due to the constraints of the bore within the MRI scanner, muscle size and moment arm measurements were also taken at a different knee joint angle to the strength measurements. These discrepancies could potentially confound the comparison of strength and morphological variables. For example, quadriceps femoris CSAs and architecture are known to change substantially between rest and maximum contraction (55). Although we have recently found LTT to have a stiffer patella tendon compared with UT, the greater strength of this group appears to produce similar muscle shortening, and thus presumably architectural changes, at MVC (56). Therefore, we are not aware of any systematic effects that might interact with these potential confounders and influence the comparison of LTT and UT groups within the current study.

Finally, the use of B-mode ultrasound presents a number of methodological issues when quantifying muscle architecture in vivo (for a review, see Ref. 36). In the present study by using a relatively long probe (92 mm vs. commonly used 40–60 mm) we were able to minimize the need for extrapolation of fascicle trajectory beyond the recorded image (typically <10% of the measured F_L_ was extrapolated). Architecture measurements were also performed in the knee isometric dynamometer with a knee angle of 115° (i.e., the same knee joint angle as the strength measurements), and this longer muscle length relative to rest explains why F_L_ was longer in the present study than in some previous reports (35, 70). However, we are conscious that ultrasound images are a 2-D representation of a complex 3-D structure and recommend that future work utilize more sophisticated 3-D techniques (e.g., diffusion tensor MRI).

In conclusion, the present study demonstrates that the larger quadriceps strength of LTT individuals was primarily due to greater muscle size with smaller differences in specific tension and moment arm, and thus muscle size was the primary explanation for the greater strength of LTT. The greater muscle volume (+56%) of LTT was due primarily to enhanced PCSA (41%), indicating more sarcomeres in parallel, although we also found convincing evidence for greater QF_L_ (+11%), indicating a modest difference in sarcomeres in series. Finally, there was no evidence for regional hypertrophy either between or along the quadriceps muscles after long-term RT.

## DISCLOSURES

No conflicts of interest, financial or otherwise, are declared by the authors.

## AUTHOR CONTRIBUTIONS

T.M.M-W, T.G.B., G.M., and J.P.F. conceived and designed research; T.M.M-W, T.G.B., and G.M. performed experiments; T.M.M-W, T.G.B., and G.M. analyzed data; T.M.M-W, T.G.B., G.M., and J.P.F. interpreted results of experiments; T.M.M-W, T.G.B., G.M., and J.P.F. prepared figures; T.M.M-W, T.G.B., G.M., and J.P.F. drafted manuscript; T.M.M-W, T.G.B., G.M., and J.P.F. edited and revised manuscript; T.M.M-W, T.G.B., G.M., and J.P.F. approved final version of manuscript.

## GLOSSARY

Θ_P_: Pennation angle
ACSA: Anatomical cross-sectional area
QACSA_MAX_: Sum of maximal anatomical cross-sectional areas
CSA: Cross-sectional area
EMG: Electromyography
F_L_: Fascicle length
HEMG_MAX_: Hamstrings EMG amplitude
IPAQ: International Physical Activity Questionnaire
KF MVT: Knee flexor maximal voluntary torque
LTT: Long term resistance trained
MRI: Magnetic resonance imaging
MVC: Maximal voluntary contraction
MVT: Quadriceps maximal isometric voluntary torque
PTMA: Patella tendon moment arm
PCSA: Physiological cross-sectional area
_EFF_PCSA: Effective physiological cross-sectional area
Q_EFF_PCSA: Sum of effective physiological cross-sectional area
Q_VOL_: Quadriceps volume
QF_L_: Mean quadriceps fascicle length
QΘ_P_: Mean quadriceps pennation angle
RT: Resistance training
RF: Rectus femoris
ST: Maximal voluntary specific tension
UT: Untrained
VI: Vastus intermedius
VL: Vastus lateralis
VM: Vastus medialis
